# No clinically significant difference in postoperative pain and side effects comparing conventional and enhanced recovery total hip arthroplasty with early mobilization

**DOI:** 10.1007/s00402-023-04858-2

**Published:** 2023-04-29

**Authors:** Jan Reinhard, Melanie Schindler, Franziska Leiss, Felix Greimel, Joachim Grifka, Achim Benditz

**Affiliations:** Department of Orthopedic Surgery, University Medical Center Regensburg, Asklepios Klinikum Bad Abbach, Kaiser-Karl-V-Allee 3, 93077 Bad Abbach, Germany

**Keywords:** Postoperative pain, Total hip arthroplasty (THA), Fast track surgery, Early mobilization, Enhanced recovery after surgery (ERAS), QUIPS

## Abstract

**Introduction:**

Enhanced recovery after surgery (ERAS) leads to less morbidity, faster recovery, and, therefore, shorter hospital stays. The expected increment of primary total hip arthroplasty (THA) in the U.S. highlights the need for sufficient pain management. The favorable use of short-lasting spinal anesthesia enables early mobilization but may lead to increased opioid consumption the first 24 h (h) postoperatively.

**Methods:**

In a retrospective study design, we compared conventional THA with postoperative immobilization for two days (non-ERAS) and enhanced recovery THA with early mobilization (ERAS group). Data assessment took place as part of the “Quality Improvement in Postoperative Pain Treatment project” (QUIPS). Initially, 2161 patients were enrolled, resulting in 630 after performing a matched pair analysis for sex, age, ASA score (American-Society-of-Anesthesiology) and preoperative pain score. Patient-reported pain scores, objectified by a numerical rating scale (NRS), opioid consumption and side effects were evaluated 24 h postoperatively.

**Results:**

The ERAS group revealed higher activity-related pain (*p* = 0.002), accompanied by significantly higher opioid consumption (*p* < 0.001). Maximum and minimum pain as well as side effects did not show significant differences (*p* > 0.05).

**Conclusion:**

This study is the first to analyze pain scores, opioid consumption, and side effects in a matched pair analyses at this early stage and supports the implementation of an ERAS concept for THA. Taking into consideration the early postoperative mobilization, we were not able to detect a difference regarding postoperative pain. Although opioid consumption appeared to be higher in ERAS group, occurrence of side effects ranged among comparable percentages.

## Introduction

Leading to less morbidity, faster recovery and, therefore, shorter hospital stays, enhanced recovery after surgery programs (ERAS) experience growing acceptance and worldwide adoption [22, 28, 35]. Being initially established for colorectal operations, ERAS gained more and more importance in orthopedic surgery [21, 23]. Big orthopedic operations such as primary total hip arthroplasty (THA) are accompanied by a resulting pathophysiologic catabolism and, therefore, long recovery. ERAS programs effectively reduce catabolism with reduced loss of muscular strength as well as less thromboembolic and gastrointestinal adverse reactions [3, 23, 24].

Due to demographic change, current predictions expect an incremental rise of primary THA in the U.S. by 284% in 2040, reaching a preliminary peak of 1.429.000 procedures annually [34]. Although primary THA was named the most successful operation of the century, about 10% of patients report postoperative dissatisfaction [16, 18, 26]. The main reason for postoperative dissatisfaction lies in chronic pain, followed by limitation of function [16]. Therefore, targeted multimodal pain management is of fundamental importance to inhibit the development of chronic pain syndromes [7, 14]. Spinal anesthesia as well as the combination of spinal and general anesthesia showed lower postoperative pain scores compared to general anesthesia [15]. Different studies report the advantages of ERAS concepts in total joint arthroplasty [11, 13, 22, 25]. A recent study compared the postoperative functional outcome and quality of life of patients receiving primary THA with an ERAS concept and such receiving conventional THA. Patients in the ERAS group reported superior functional results in the WOMAC score 1 year postoperatively [27]. Nevertheless, literature review reveals inconsistent data availability concerning early postoperative pain [17, 19, 39]. While many studies aim on reduction of the length of hospital stay as the main indicator for success, climaxing in same day discharge, the early pain, and complications 24 h postoperatively can mostly not be evaluated due to early discharge [19, 39].

The beneficial use of short-lasting spinal anesthesia in ERAS concepts enables early mobilization [22]. Due to its short-lasting effect with efficacy for only up to 2–4 h, one might expect a higher oral opioid consumption in the first 24 h postoperatively which may result in increased rates of side effects. Even though some studies report lesser opioid consumption in fast-track surgery, no study compared opioid consumption and occurrence of side effects in a matched pair analyses at that early stage [31].

## Aim of the study

In a matched pair analysis of 630 patients, comparing conventional with enhanced recovery THA with early mobilization, we aimed to evaluate patient-reported pain, opioid consumption, and side effects 24 h postoperatively.

## Methods

Data assessment took place between 2009 and 2021. In a retrospective study design, in total, 2161 patients receiving primary THA at our university hospital were included. Criteria for inclusion were cementless primary THA and fully orientated patients, older than 18 years. Patients with BMI > 40 kg/m^2^, immobilization in a wheelchair or need for a wheeled walker were excluded. Furthermore, refusal to participate, disorientation, sedation, visitors during data assessment or cognitive dysfunction represented criteria for exclusion. An independent, special trained pain nurse interviewed the patients 24 h postoperatively. A validated 16-item questionnaire was used, asking for minimal and maximum pain since surgery on a numerical rating scale (NRS, 0 = no pain, 10 = worst imaginable pain) to document postoperative pain management and occurrence of complications. Satisfaction with pain management and participation in pain management was objectified by an inversed NRS (0 = completely dissatisfied, 10 = perfectly satisfied). The file reporting demographic data as well as the patients’ questionnaire is provided under the following URL: https://www.quips-projekt.de/services/dateien and attached to the document.

Patients in both groups received primary THA via a modified Watson–Jones approach without transection of muscular tissue [6]. Patients were placed in lateral position and an anterolateral mini-incision was performed. Using the intermuscular plane between tensor fascia lata and gluteus medius, the integrity of the muscles is preserved while the intactness of the posterior capsule prevents posterior dislocation [6]. Postoperative pain management was identical in both groups based upon WHO three-step analgesic ladder [1, 8]. Consisting of three steps which range from mild to severe pain, it is well established and widely used. First step describes mild pain and is treated by nonsteroidal anti-inflammatory drugs (NSAIDs) without adjuvants. In step two, additional weak opioids are used, while severe pain, representing step three, is treated by additional potent opioids [1, 8].

The established ERAS concept for primary THA consisted of local infiltration analgesia, special anesthesia, and a targeted postoperative treatment protocol: Patients received education before the operation in terms of pain management and gait training by physiotherapists. They were trained to walk with crutches in advance and educated what the limitations in mobilization they have to expect directly after the operation. One hour preoperatively, participants were administered a non-steroid-anti-inflammatory-drug (etoricoxib 90 mg). Every patient received a short-lasting spinal anesthesia (4 ml prilocaine 1%, hyperbaric and 10 µg sufentanil) combined with the intravenous application of dexamethasone (8 mg). In addition, patients received tranexamic acid intravenously (1 g) as well as topically (2 g). Intraoperatively local infiltration analgesia (ropivacaine 200 mg, adrenaline 0.5 mg) was administered periarticular, femoral, acetabular, and subcutaneously. No drains were placed in the operating field. Patients were immediately allowed full weight-bearing and first mobilization was carried out 2–3 h postoperatively at an Intermediate Care Unit (IMC). Every patient received intensive physiotherapy by a special trained physiotherapist for half an hour two times a day. Patients were encouraged to use a special developed exercise course with the aim to improve range of motion and balance.

In contrast to the ERAS concept, patients receiving conventional primary THA (non-ERAS) received neither gait training, nor analgesic medication directly before the operation. A long-lasting spinal anesthesia was performed (4 ml bupivacaine, 0.5%). Intraoperatively, neither local infiltration analgesia, nor tranexamic acid or dexamethasone was administered. Wound drains were consequently applied. At the earliest, patients were mobilized for the first time the day after the operation. Postoperative partial weight-bearing was allowed. Physiotherapy took place once a day.

### Data assessment

Data acquisition was performed as part of the Quality Improvement in Postoperative Pain Treatment project (QUIPS), a nationwide German benchmarking initiative for postoperative pain [30]. Including data sets from over 600.000 patients and over 200 participating hospitals, it demonstrates the largest database for acute postoperative pain worldwide. The project is supported by the German Society of Anesthesiologist and the German Society of Surgeons [29]. All data were anonymized.

The present study was conducted in agreement with the ethical standards of the Declaration of Helsinki (1975). A member of the study crew informed every patient orally as well as in written form about the study. Every participant signed the informed consent before enrollment. Participation was voluntary with possible withdrawal at any time. The ethics committee as well as the data security board of the Jena University Hospital (Jena, Germany) approved the data acquisition for the QUIPS project. The study is registered in the DRKS with number DRKS00006153 (WHO register).

### Statistical analysis

To ensure groups of identical size and cofounders, we performed a 1:1 matched pair analysis, based upon gender, age, ASA, and chronic NRS preoperatively [27]. If there was more than one possible matching partner, the matching patient was chosen randomly. Metric variables are noted as median ± interquartile range (IQR). Categorical variables are noted in relative frequencies. Shapiro–Wilk Normality Test was used to test for normal distribution. Data were not normally distributed. To test for statistical significance, we used C*hi-square test* and *nonparametric Mann–Whitney U*. Statistical significance was considered at *p* < 0.05. Statistically significant data are noted in italics. Statistical analysis was performed with SPSS (IBM SPSS Statistics 28, International Business Machines Corporation (IBM), Armonk, New York, U.S.).

## Results

**Fig. 1:**
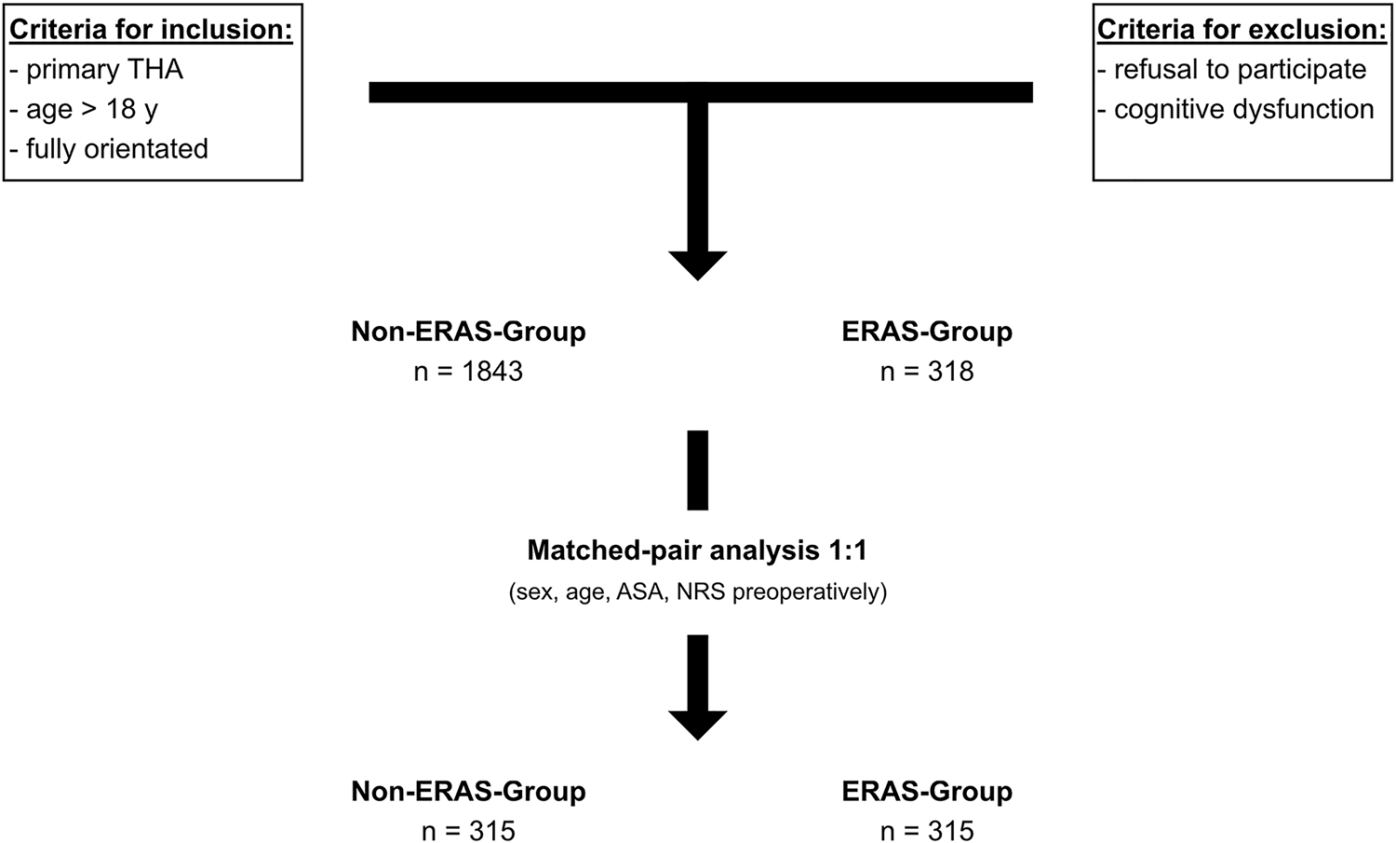
Flowchart methods

Between 11/2009 and 11/2021, 1843 patients received conventional primary THA, while 318 patients received THA featuring the described enhanced recovery concept. After performing a 1:1 matched pair analysis for gender, age, ASA, and NRS preoperatively, each group consisted of 315 patients. The flowchart is illustrated in Fig. 1.

As result of the matched pair analysis, demographic data of gender, age, ASA, and preoperative pain are almost identical. The duration of surgery appeared to be significantly shorter in the ERAS group than in conventional THA (*p* = 0.002, Table 1). Occurrence of chronic pain preoperatively did not reveal significant differences. Significantly more patients in Non-ERAS group had chronic pain not only in the operated region (*p* = 0.032). Demographic data are noted in Table 1.

**Table 1:** Demographic data of the 630 included patients

	Non-ERAS (315)				ERAS (315)				*p* value
	Median ± IQR		Range		Median ± IQR		Range		
Age (years)	65 ± 20		25–95		65 ± 20		25–95		0.99
Sex (male: female)	180:135				180:135				0.99
Duration of surgery (min)	*65* ± *20*		*36–151*		*61.5* ± *16*		*26–121*		*0.002*
ASA Score frequency (%)	1	2	3	4	1	2	3	4	0.99
	28.6	63.8	7.6	0	28.6	63.8	7.6	0	
Chronic pain > 3 months before surgery									
- Operated region (%)	*94.1*				*97.4*				*0.032*
- Operated + 1 other region (%)	*5.9*				*2.3*				
NRS chronic pain	7 ± 3		2–9		6 ± 3		2–9		0.23
Opioid preoperatively (%)	4.2				3.2				0.672

Values in italics represent a significant difference between the two groups (*p* < 0.05)

**Fig. 2:**
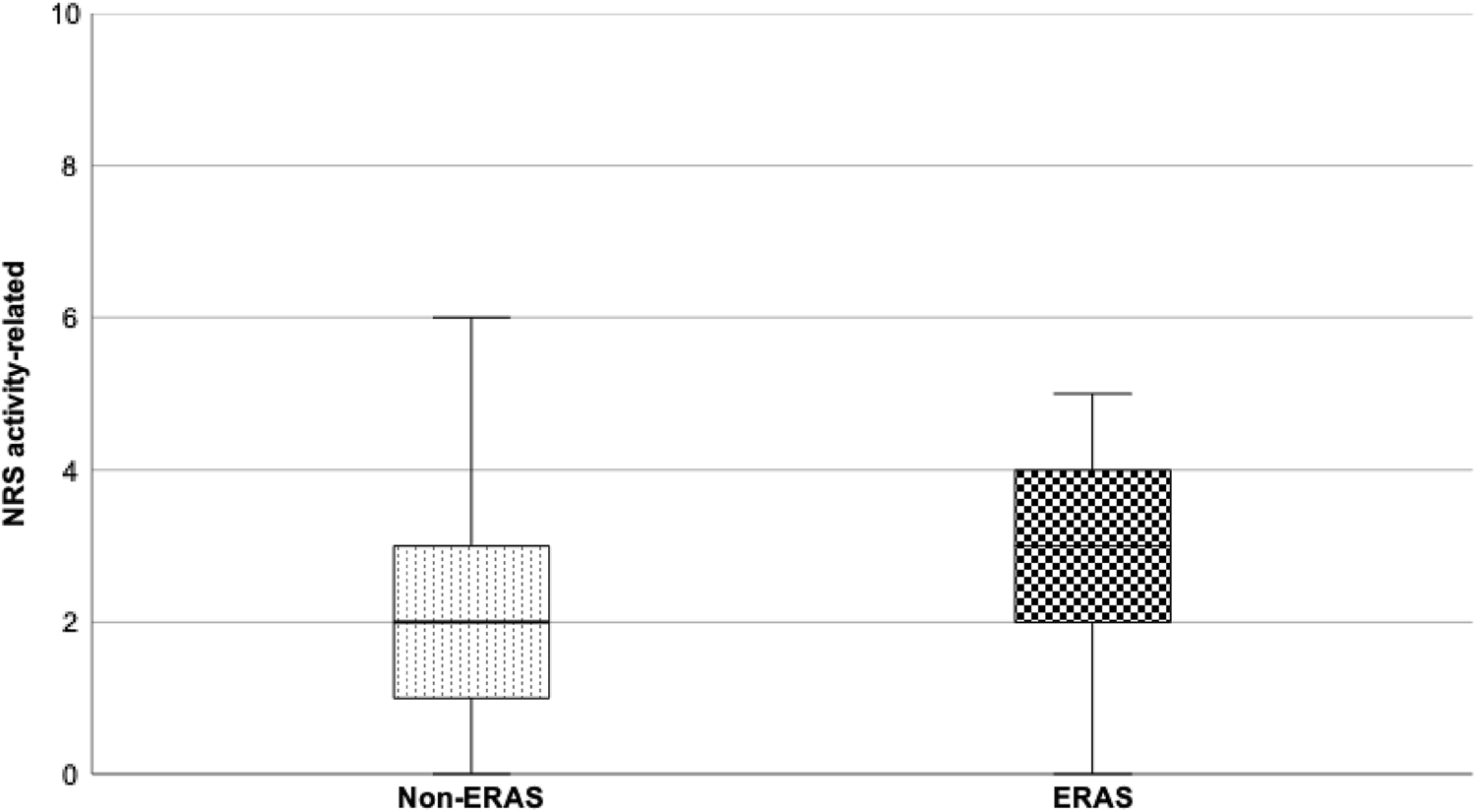
Boxplot activity-related pain 24 h postoperatively

### Pain development and functional outcome

Evaluation of minimum and maximum pain as well as satisfaction and patients’ participation in pain management did not show significant differences between the two concepts 24 h postoperatively (*p* > 0.05, Table 2*)*. The ERAS group revealed significantly higher pain scores in activity-related pain (*p* = 0.002, Table 2). Figure 2 shows the boxplots of the two groups for activity-related pain.

**Table 2:** Mean NRS minimum, maximum, and activity-related pain, satisfaction, and participation in pain management 24 h postoperatively

	Non-ERAS		ERAS		*p* value
	Median ± IQR	Range	Median ± IQR	Range	
NRS minimum (min.)	0 ± 0	0–1	0 ± 0	0–1	0.725
NRS maximum (max.)	5 ± 2	0–9	5 ± 2	0–9	0.178
NRS activity-related	*2* ± *2*	*0–6*	*3* ± *2*	*0–5*	*0.002*
Satisfaction	10 ± 0	6–10	10 ± 0	3–10	0.247
Participation	10 ± 0	6–10	10 ± 0	8–10	0.307

Values in italics represent a significant difference between the two groups (*p* < 0.05)

**Fig. 3:**
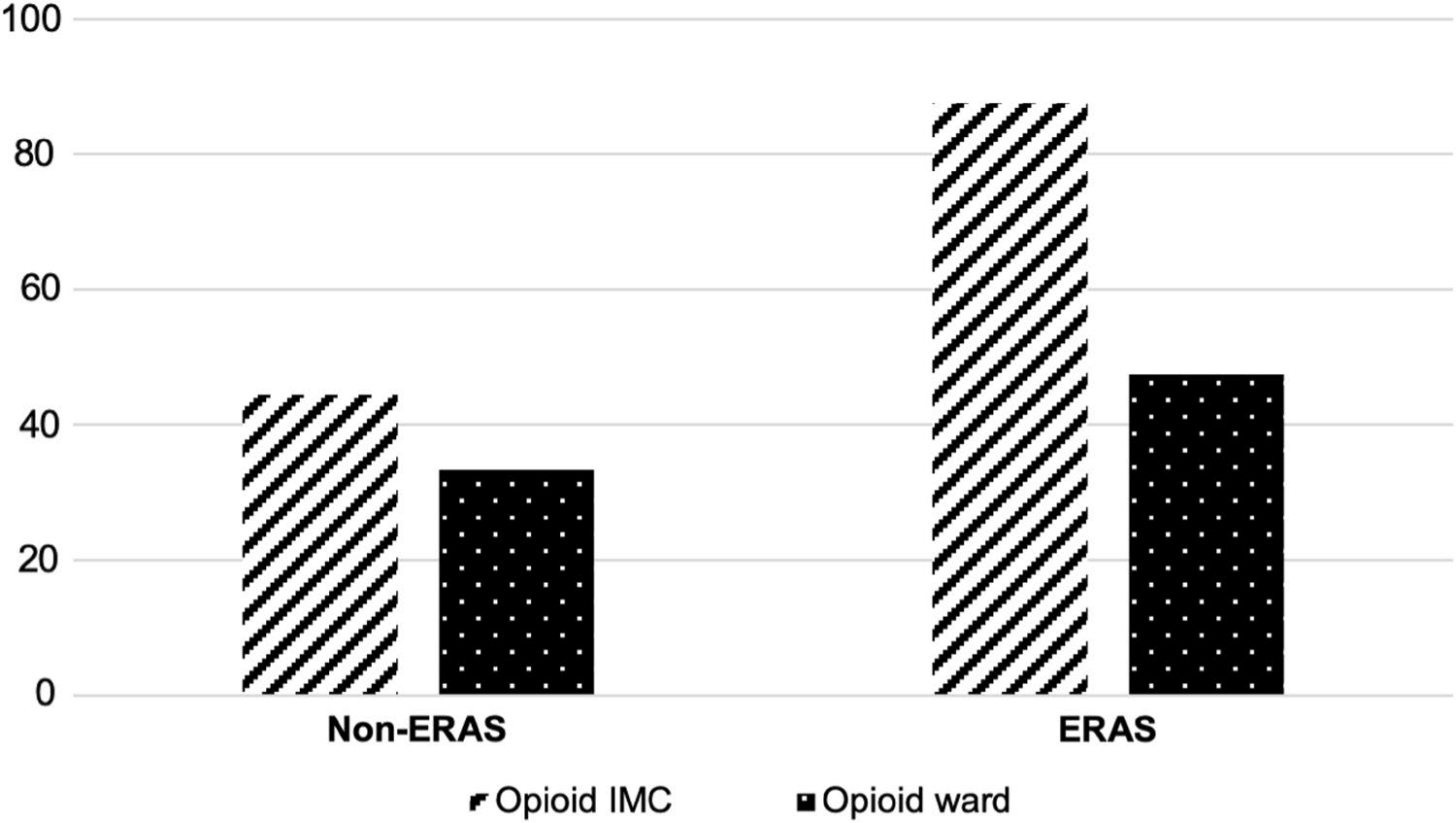
Opioid consumption (%) at Intermediate Care Unit (IMC) and ward 24 h postoperatively

The functional outcome revealed identical results and the ERAS group showed a significantly higher percentage of pain affected ability to move (*p* = 0.019). The other functional outcome parameters ranged among comparable values in both groups (*p* > 0.05, Table 3).

**Table 3:** Functional outcome after surgery 24 h postoperatively

	Non-ERAS	ERAS	*p* value
Pain affected ability to move (%)	*26.0*	*34.9*	*0.019*
Pain affected ability to cough/take a deep breath (%)	5.1	6.7	0.498
Pain affected ability to sleep (%)	9.8	10.8	0.794
Pain affected mood (%)	0.6	1.3	0.686

Values in italics represent a significant difference between the two groups (*p* < 0.05)

### Oral opioid consumption and side effects

Patients in the ERAS group revealed significantly higher demand for oral opioids at the IMC unit as well as at ward than the conventional THA group (both *p* < 0.001). The data are noted in Table 4. Figure 3 illustrates the comparison between both groups in regards to the relative frequency of opioid consumption.

**Table 4:** Oral opioid consumption at Intermediate Care Unit (IMC) and hospital ward 24 h postoperatively

	Non-ERAS	ERAS	*p* value
Opioid consumption IMC (%)	*44.4*	*87.6*	< *0.001*
Opioid consumption ward (%)	*33.4*	*47.5*	< *0.001*

Values in italics represent a significant difference between the two groups (*p* < 0.05)

The evaluation of the occurrence of side effects did not show significant differences between both groups (*p* > 0.05, Table 5).

**Table 5:** Occurrence of side effects 24 h postoperatively

	Non-ERAS	ERAS	*p* value
Nausea (%)	25.1	24.1	0.853
Dizziness (%)	28.3	32.5	0.298
Tiredness (%)	21.0	24.4	0.342

## Discussion

This study is the first to analyze pain scores, opioid consumption, and side effects after primary THA at this early stage, comparing conventional THA and an ERAS concept. Although opioid consumption and pain scores were significantly higher within 24 h after surgery, it supports the implementation of an ERAS concept as the advantages like less morbidity and faster recovery predominate.

The comparison between conventional THA and an ERAS concept with early mobilization revealed significantly higher activity-related pain as well as higher opioid consumption in the ERAS group the first 24 h postoperatively. It must be taken into consideration that patients receiving conventional THA were first mobilized more than 24 h after surgery, accordingly after the data assessment. Hence, patients in the conventional THA group did not experience early mobilization and, therefore, did not undergo activity-related pain the first 24 h. In contrast, patients in the ERAS group were exposed to early mobilization as early as 2 h after surgery at an IMC unit. The pain scores after 24 h postoperatively were not imposed; therefore, it is not possible to make a final statement on further opioid usage. Nevertheless, the opioid usage on ward was significantly less than on IMC.

In addition, although the ERAS group reported significantly more activity-related pain, the difference is not that big and might be from minor clinical importance (NRS activity related: ERAS 3 ± 2 vs. non-ERAS 2 ± 2). Farrar et al*.* defined a change of at least 2 points on an NRS as clinically relevant [12]. Therefore, a statistically significant difference of one point on an NRS might be of minor clinical relevance for the patients. This assumption is indirectly supported by absence of a significant difference in maximum and minimum pain between the two groups (both *p* > 0.05). In summary, taking into consideration the early postoperative mobilization, we were not able to detect a difference regarding postoperative pain. Even though there is statistically difference it might not be clinically different.

Compared to conventional THA, opioid consumption in ERAS group at IMC unit almost doubled. At ward, opioid demand still appeared to be higher than in the non-ERAS group, though less distinctive. One explanation might be the long-lasting effect of opioid spinal anesthesia in conventional THA, in contrast to the short-lasting one in the ERAS group. While the difference is more pronounced at IMC unit, the effect of anesthesia is fading, and it already harmonized more at ward. As described above, the additional stress and pain through early mobilization in the ERAS group have to be taken into consideration and might represent the true cause for the measured difference.

Another reason for the high opioid consumption within the first 24 h postoperatively may be the occurrence of rebound pain after regional anesthesia. The phenomenon of rebound pain as a side effect of regional anesthesia was first reported in 2005; nevertheless, its causes are still mainly unclear [37]. It is described as a disproportional excessive pain as the effect of local anesthesia fades away after primary good perioperative pain compensation [36, 37]. It is discussed controversially in literature with prevalence of severe rebound pain in up to 40% of patients [10, 38]. Despite its high occurrence, studies report high rates of patient satisfaction [4]. In an analysis of possible prevention strategies, Dawson et al. supported a multimodal therapy concept with wider use of opioids [10].

Some studies report a significantly higher occurrence of dizziness, nausea, and vomiting in patients receiving fast-track surgery [2, 20]. In contrast, the evaluation of postoperative side effects did not show significant differences between both groups. Even though opioid demand appeared to be significantly higher in the ERAS group, possible side effects such as nausea, vomiting, and tiredness showed comparable values.

The duration of surgery was significantly shorter in the ERAS group (*p* = 0.002). Extended surgery time demonstrates a major risk factor for infections and postoperative complications like delirium [32, 33]. Moreover, operation time is expensive, representing an economical factor to reduce hospital costs [9]. Nevertheless, reduction of surgery time should not be seen as the primary goal, according to the fast-track protocol principle “first better—then faster” [40, 41]. Nevertheless, we were able to disprove the fear of an extended surgery time through additional interventions such as generous local infiltration anesthesia in an ERAS concept. This study did not focus on shortening the hospital stay or on evaluating the readmission rate, as it has been already described, and proved in several publications [5, 22, 25].

The present study features some limitations. First, the study followed a retrospective design. For better comparison we performed a matched pair analysis for sex, age, ASA, and preoperative pain scores to reduce covariate bias. Second, data acquisition only took place at one instance without further assessments and the evaluation of progress is thus missing with regards to the QUIPS-projects study design. Third, there may be a potential selection bias through the novel establishment of the ERAS concept at our university hospital.

## Conclusion

Although the biggest differences in pain and opioid consumption are expected the first 24 h after the operation, the present study is the first to analyze this in depth in a matched pair analysis. Taking into consideration the early mobilization, we did not detect a difference in postoperative pain comparing conventional THA and THA with an ERAS concept. Although opioid consumption appeared to be significantly higher in the ERAS group, the occurrence of side effects ranged among comparable percentages. In addition, the operation time in the ERAS group was significantly shorter. In summary, this study supports the implementation of an ERAS concept for primary THA.

## Data Availability

On request, data are available at the authors’ institution.
